# Spectrum of *MEFV* Variants and Genotypes among Clinically Diagnosed FMF Patients from Southern Lebanon

**DOI:** 10.3390/medsci8030035

**Published:** 2020-08-17

**Authors:** Ali El Roz, Ghassan Ghssein, Batoul Khalaf, Taher Fardoun, José-Noel Ibrahim

**Affiliations:** 1 Faculty of Public Health, Lebanese German University (LGU), Sahel Alma 25136, Lebanon; a.elroz@lgu.edu.lb (A.E.R.); g.ghssein@lgu.edu.lb (G.G.); batoul.khalaf95@gmail.com (B.K.); 2 Mashrek Medical Diagnostic Center, Tyre 62111, Lebanon; taher.fardoun@gmail.com

**Keywords:** FMF, South Lebanon, variants, prevalence

## Abstract

**Background:** Familial Mediterranean Fever (FMF) is an autosomal recessive auto-inflammatory disease characterized by pathogenic variants in the *MEFV* gene, with allele frequencies greatly varying between countries, populations and ethnic groups. **Materials and methods:** In order to analyze the spectrum of *MEFV* variants and genotypes among clinically diagnosed FMF patients from South Lebanon, data were collected from 332 participants and 23 *MEFV* variants were screened using a Real-Time PCR Kit. **Results:** The mean age at symptom onset was 17.31 ± 13.82 years. The most prevalent symptoms were abdominal pain, fever and myalgia. *MEFV* molecular analysis showed that 111 patients (63.79%) were heterozygous, 16 (9.20%) were homozygous, and 47 (27.01%) carried two variants or more. E148Q was the most encountered variant among heterozygous subjects. E148Q/M694V was the most frequent in the compound heterozygous/complex genotype group, while M694I was the most common among homozygous patients. Regarding allele frequencies, M694V was the most common variant (20.7%), followed by E148Q (17.1%), V726A (15.7%) and M694I (13.2%). **Conclusion:** The high percentage of heterozygous patients clinically diagnosed as FMF highlights the pseudo-dominant transmission of the disease in Lebanon and emphasizes the importance of molecular testing for a more accurate diagnosis and better management and treatment of FMF.

## 1. Introduction

Familial Mediterranean Fever (FMF) is an autosomal recessive auto-inflammatory disease caused by pathogenic variations in the *MEFV* (for MEditerranean FeVer) gene, located on the short arm of chromosome 16 (16p13.3). This gene is composed of 10 exons and 781 codons with two mutational hot spots at exon 2 and exon 10 [1]. The *MEFV* gene encodes for the pyrin protein, which is a part of the pyrin inflammasome, a cytoplasmic multiprotein organelle involved in the production of biologically active IL-1. Mutated forms of pyrin result in the impaired assembly of the inflammasome, thus leading to the excessive production of pro-inflammatory cytokines, mainly IL-1β, which plays a central role in the pathogenesis of FMF [2]. 

FMF is characterized by periodic fever episodes accompanied by sterile arthritis, pleurisy, peritonitis, rashes, pericarditis and/or erysipelas-like erythema. The frequency of the crises is variable and may occur in response to a stimulus such as emotional stress, extensive physical activity or menstruation or without any recognized triggering factor [1]. The most efficient treatment for FMF is colchicine. If untreated, FMF can worsen and evolve to cause renal amyloidosis, which can end up causing renal failure [1]. Even though populations descending from the Mediterranean basin including Arabs, Turks, Armenians, Jews and Italians carry the highest risk of having FMF [3,4,5,6,7], sporadic cases in the Far East have also been reported [8]. 

More than 370 *MEFV* gene sequence variants have been reported to date and classified into five categories, namely, benign, likely benign, pathogenic, likely pathogenic and variants of uncertain significance (VUS) [9]. The most common variants reported among FMF patients are M694V, M694I, V726A and M680I in exon 10 as well as E148Q in exon 2. These five variants are known to be responsible for about 85% of cases [10]. Despite these being the most common, the allele frequency of these variants varies not only between countries but also between populations, communities, ethnic groups and religions [11]. Indeed, the M694V pathogenic variant is the most common among Arab and non-Arab populations except for the Ashkenazi Jews and non-Arab Iraqis, where V726A is the most predominant [12]. On the other hand, M694I is more particularly observed among Egyptian and Algerian populations while M680I is more frequent in Tunisia [13,14,15,16].

Previous research in the literature analyzed the prevalence of the most common *MEFV* gene variants among unrelated Lebanese patients [11,17,18]. The present study aims to analyze the frequency of a larger spectrum of *MEFV* gene variants and genotypes in a cohort of patients from Southern Lebanon presenting a clinical diagnosis of FMF.

## 2. Materials and Methods 

### 2.1. Study Design and Population

The study was carried out over a period of three years, between 2014 and 2017. The research involved 332 Lebanese subjects clinically diagnosed as having FMF according to Heller Criteria [19] and referred by their physician to the Mashrek Diagnostic Center for *MEFV* gene testing. Socio-demographic characteristics, clinical symptoms and molecular analysis data were collected for all participants. A written informed consent was obtained from all subjects prior to enrollment.

### 2.2. Variant Analysis

A blood sample was collected from all participants in EDTA tubes and DNA was extracted from white blood cells using the FMF Multiplex Real Time PCR Kit (SNP-Biotechnology, Ankara, Turkey). PCR amplification was then carried out according to the manufacturer’s instructions. Exons 1, 2, 3, 5 and 10 were amplified by a PCR program set as follows: 95 °C denaturation for 30 s, 55 °C hybridization for 30 s and 72 °C elongation for 30 s.

The 23 gene variants analyzed with the kit were the following: E084K in exon 1; E148Q, E148V, E167D, E230Q, E230K, P283L, R202Q, T267L, L110P and G304R in exon 2; P369S and R408Q in exon 3; F479L in exon 5; and M694V, M694I, M680IG/C, M680IG/A, I692del, V726A, A744S, K695R and R761H in exon 10. 

## 3. Results

Out of the 332 subjects tested for FMF, 161 were females and 171 were males. In 65.94% of cases, the age of onset of the first symptoms was before 20 years old, with a mean value of 17.31 ± 13.82 years. The most prevalent symptom observed in our sample was abdominal pain, being present in 95% of the patients, followed by fever (50%) and myalgia (46%) (Table 1). 

The molecular analysis of the *MEFV* gene variants revealed a negative result in 144 (43.37%) participants, while 188 (56.63%) subjects carried at least one *MEFV* variant. The distribution of the most prevalent variants and their allele frequencies among the 188 subjects is represented in Table 2. M694V was ranked as the most common variant, accounting for 20.6% of cases. E148Q was found in 17.9% of cases, followed by V726A, R202Q, M694I and A744S, accounting for 16.0%, 12.6%, 11.8% and 10.7%, respectively. Moreover, M694V had the highest allele frequency (20.7%), followed by E148Q (17.1%), V726A (15.7%), M694I (13.2%), R202Q (11.8%) and, finally, A744S (10.0%). 

The benign/likely benign polymorphisms detected in the present study—namely, R202Q and G304R—cannot be responsible for FMF and hence were not included in the classification of patients according to allele status. Out of the 174 remaining patients carrying FMF-causing variants, 111 (63.79%) were found to be heterozygous (one single variant), 16 (9.20%) were homozygous and 47 (27.01%) carried multiple *MEFV* variants, hence considered as compound heterozygous (variants on both alleles) or having a complex genotype (variants on the same allele). The male to female ratio in the FMF patients with *MEFV* variants was 1.4 (58.45% vs. 41.55%), and the mean age of the first symptoms was 18.15 ± 14.19 years. The distribution of the age at symptom onset in these patients is represented in Table 3.

Table 4 represents the classification of clinically diagnosed FMF patients according to allele status. The results showed that in heterozygous patients, the most encountered variant was E148Q, affecting 16.1% of the 174 enrolled patients. On the other hand, the E148Q/M694V genotype was the most frequent in the compound heterozygous/complex genotype group, being responsible for 5.8% of cases. Finally, the M694I pathogenic variant was the most common among homozygous patients, affecting 2.3% of patients.

## 4. Discussion

### 4.1. Age of Diagnosis and Gender Distribution

The present data are in line with previous studies showing that the majority of FMF patients are diagnosed before 20 years old [1,10,20]. On the other hand, the noticeably small percentage of patients diagnosed at an age below one is expected and was in agreement with published reports suggesting that infants at such an age have insufficient verbal abilities for the diagnosis to be made [20]. Furthermore, the late diagnosis of patients above 40 is common in endemic regions. This might be attributed to misdiagnosis or misinterpretation by the physician, and it is mostly due to the acute and unpredictable nature of the FMF attack [21]. 

The overall male to female ratio in our cohort of FMF patients was 1.4, which is in accordance with previous studies reporting that FMF affects men more than women with an approximate ratio of 3/2 (1.5) and attributing this finding to the lower penetrance of the disease in women [12,18,22,23]. Other possible hypotheses explaining this result include the greater resistance of females to pain and the possibility of confusing abdominal and gynecological pain, hence contributing to the underdiagnosis or misdiagnosis of the disease [18]. However, further studies are necessary to understand the exact mechanism underlying the variability in FMF susceptibility between males and females.

### 4.2. Benign Polymorphisms and Variants of Uncertain Significance (VUS)

The study of the most common variants observed in our sample of FMF patients revealed that E148Q was the most frequent in heterozygous carriers. Interestingly, a previous study conducted by Medlej-Hashim et al. in 2005 on a group of 100 Lebanese healthy unrelated individuals showed that 5% of tested chromosomes carried the E148Q variant, hence supporting the idea of E148Q being a benign polymorphism rather than a pathogenic variant [24]. Indeed, researchers have found that patients homozygous for E148Q had milder symptoms and a delayed disease onset than patients homozygous for other *MEFV* variants [25]. On the other hand, our findings demonstrated the presence of E148Q in 18 patients carrying two *MEFV* variants. The genotype E148Q/M694V observed in 10 patients was previously reported by Touitou et al. as a complex allele rather than a compound heterozygous genotype [10]. Accordingly, a study conducted on 233 patients of Sephardic Jewish origin living in France and 213 disease-free relatives of these patients showed a comparable frequency of the E148Q/M694V genotype between the two groups, thus decreasing the probability of E148Q being an FMF-causing variant [26].

Other variants detected at a high rate in the study population and known to have a benign effect or an uncertain clinical significance include R202Q, A744S and R408Q. Several guidelines and tools have been recently developed to improve the classification of many unsolved, unclassified or of-uncertain-significance *MEFV* gene variants [27,28]. This could result in a more accurate interpretation of the clinical consequences of *MEFV* gene variants, and in better genetic counselling and patient management.

### 4.3. Pseudo-Dominant Inheritance

Out of the 332 Lebanese subjects included in the research, 111 patients with classical FMF symptoms and admitted to the laboratory for genetic testing turned out to be heterozygous. These data emphasize the idea of the presence of FMF-like symptoms in patients carrying a single *MEFV* allele variant, despite the autosomal recessive inheritance of the disease. Indeed, previous studies described cases of an FMF pseudo-dominant transmission [10,29] that can be explained by two interpretations. The first is that a heterozygous patient may have carrier parents and therefore may have two different variants. However, the fact that most available kits do not cover all variants may give false negative results and subsequently affect the diagnosis of the disease. This case is very common in populations such as that in Lebanon where consanguineous marriage is reported at high rates [30]. The second is that some heterozygous patients may carry a severe pathogenic (high penetrance) variant such as M694V or M694I that may be present in cases of autosomal dominant transmission [29] and therefore responsible for the presence of FMF symptoms in subjects with only one identified variant [31]. Indeed, a few cases of dominantly inherited FMF have been recently reported in the literature. For example, Rowczenio et al. identified the presence of the M694del variant associated with autosomal dominantly inherited FMF in 21 patients from North Caucasus [32]. Interestingly, all patients exhibited typical FMF symptoms but with a delayed onset of the disease (median age of 18 years) and a similar response to colchicine treatment as compared to classical recessive FMF patients. Other research conducted by Procopio et al. in 2018 on a sample of 107 clinically diagnosed FMF patients revealed the presence of nine distinct variants and the association of M694V and M680I with the most severe clinical phenotype [33]. Notably, 85.98% of the patients showed a heterozygous genotype, and no significant difference in the clinical phenotype was noted between heterozygous, homozygous and compound homozygous subjects, further supporting the evidence that, contrary to recessive autosomal inheritance, heterozygous patients fulfill the criteria of clinical FMF. Taken together, these data strongly suggest that a single-gene recessive model of inheritance is incapable of fully describing the broad spectrum of *MEFV*-associated phenotypes. The existence of a “non-classic” autosomal recessive inheritance or a pseudo-dominant autosomal inheritance with incomplete penetrance and variable expressivity cannot be excluded in FMF.

### 4.4. Comparison with Data from Other Countries

The distribution of *MEFV* spectrum variants among different populations is subject to many variations, mostly related to ancestry, ethnicity and the different religious groups within the studied society. Table 5 shows the distribution of the six most common *MEFV* variants in the present study as compared to that in other populations including Arabs and non-Arabs.

Out of the 23 variants scanned, six were encountered the most in the Lebanese population, with M694V being the most frequent (20.6%), followed by E148Q (17.9%) and V726A (16.0%). R202Q, M694I and A744S were less common, with allele frequencies of 12.60%, 11.8% and 10.7% respectively. When these results were compared with data obtained from other Arab and non-Arab populations, we noticed that the R202Q gene variant was only mentioned in this study and in research performed on the Turkish population (Table 5). 

The comparison of data from the present research with studies conducted previously in Lebanon [11,17,18] revealed that M694V was found to be the leading variant in all of them. Slight differences exist concerning the allele frequencies of other variants, especially E148Q, which was the second most common variant in the present study (17.9%) while being fourth in the other ones, with allele frequencies of 8.3% and 10.1%, respectively. This can be related to the mosaic nature of the Lebanese society in terms of ethnicity, religious groups and regional distribution.

Studies conducted on other Arab populations [13,16,18,34,35,36] showed that M694V was also the most frequent in Tunisia, Morocco, Syria, Jordan and Palestine, with allele frequencies varying between studies. The notably high prevalence of the V726A pathogenic variant in Syria, Jordan and Palestine as well as in Lebanon emphasizes the findings of Touitou et al. in 2001 associating this variant with oriental Arabs [10].

On the other hand, the Egyptian population exhibited outcomes different from those in the Asian Arabic countries, where E148Q and M694I were the most common variants detected in FMF patients [22,37]. Published data in Algeria [15] also showed relevant results, with M694I ranking first with an allele frequency of 50%. This is in accordance with a report published by Samuel et al. [38] that described M694I as a pathogenic variant specific to North African Arabs.

Finally, concerning the non-Arab subjects, a study conducted by Coşkun et al. in 2015 in Van province, the Easter region of Turkey, showed that M694V was the most frequently observed variant (36.5%) among 1058 pediatric patients with suspected FMF [39]. On the other hand, Gunesacar et al. showed that R202Q was the most common variant (21.35%) in a sample of 1000 clinically diagnosed patients from Hatay Province, the Mediterranean region of Turkey, while M694V ranked third and accounted for 7.95% of cases [40]. The difference in variant frequency may be due to the heterogeneity of the study populations and the wide variety of research designs, as well as the geographic determinants and socio-demographic characteristics of the participants. Two studies conducted on Iranian populations revealed that M694V was the most common variant among FMF patients, with allele frequencies of 42.4% and 40.19%, respectively [41,42].

## 5. Conclusions

This study analyzed the mutational spectrum of *MEFV* variants among clinically diagnosed FMF patients from Southern Lebanon. The most commonly observed manifestations were abdominal pain (95%), fever (50%) and myalgia (46%). The molecular analysis of the *MEFV* variants showed that, similar to other studies conducted in Lebanon, M694V was the most frequent variant, followed by E148Q and V726A. Out of the 174 patients carrying FMF-causing variants, 111 (63.79%) turned out to be heterozygous, hence supporting the pseudo-dominant transmission of the disease in this Lebanese region where consanguineous marriage is high. Moreover, the high percentage of E148Q in heterozygous patients reinforces the hypothesis that E148Q is probably not a pathogenic variant but rather a benign polymorphism. Although two *MEFV* variants were detected in 63 patients, only 16 (9.20%) were found to be homozygous and genetically diagnosed with FMF. Testing the parents of the remaining patients is essential to determine if they are compound heterozygous before confirming the molecular diagnosis of the disease. Finally, future studies are important to screen for other rare and newly identified variants and to analyze those of uncertain significance in order to establish a more accurate diagnosis of FMF, and hence provide patients with a personalized treatment and better management of the disease outcomes.

## Figures and Tables

**Table 1:** Distribution of the most common symptoms among patients.

Clinical Manifestation	Number	Percent
Abdominal pain	315	95%
Fever	166	50%
Myalgia	152	46%
Joint pain	66	20%
Chest pain	33	10%
Nausea	26	8%
Diarrhea	23	7%
Vomiting	16	5%

**Table 2:** Distribution of *MEFV* variants and allele frequencies among participants.

	Variant Classification *	Variants Frequency N (%)	Allele Frequency N (%)
M694V	Pathogenic	54 (20.6)	58 (20.7)
E148Q	Uncertain significance	47 (17.9)	48 (17.1)
V726A	Pathogenic	42 (16.0)	44 (15.7)
R202Q	Benign	33 (12.6)	33 (11.8)
M694I	Pathogenic	31 (11.8)	37 (13.2)
A744S	Uncertain significance	28 (10.7)	28 (10.0)
R408Q	Uncertain significance	7 (2.7)	10 (3.6)
R761H	Likely pathogenic	6 (2.3)	8 (2.9)
Others		14 (5.4)	14 (5.0)

* Based on Infevers database for auto-inflammatory diseases.

**Table 3:** Distribution of the age at symptom onset in patients with *MEFV* variants.

Age (Years)	Percentage
≤1	2.45%
[1–20]	58.89%
[20–40]	28.22%
[40–55]	10.44%

**Table 4:** Frequency of the most common *MEFV* variants and genotypes of Familial Mediterranean Fever (FMF) patients.

Variant	Genotype	Number	Percent
Heterozygous (N = 111) (63.79%)	E148Q	28	16.1
	M694V	25	14.4
	V726A	20	11.5
	A744S	16	9.2
	M694I	10	5.8
	Others	12	6.9
Compound Heterozygous/Complex genotype (N = 47) (27.01%)	M694V/E148Q	10	5.8
	M694V/V726A	9	5.2
	V726A/A744S	5	2.9
	M694I/E148Q	4	2.3
	M694I/A744S	4	2.3
	Others	15	8.7
Homozygous (N = 16) (9.20%)	M694I	4	2.3
	M694V	2	1.1
	M694V-M694I	2	1.1
	V726A	2	1.1
	R408Q	2	1.1
	R761H	2	1.1
	Others	2	1.1
Total		174	100

**Table 5:** Distribution and frequency of the six most common *MEFV* variants in Arab and non-Arab populations.

Country/Population	Variants (%)					
	M694V	E148Q	V726A	R202Q	M694I	A744S
Current study/332	20.6	17.9	16.0	12.6	11.8	10.7
Lebanon [18]/558	30.3	8.3	19.4	-	12.8	1.2
Lebanon [43]/376	28.9	10.1	19.3	-	12.1	-
Jordan [18]/78	34.6	6.4	19.2	-	2.6	-
Jordan [16]/3959	30	21.5	20	-	8.3	3.1
Syria [34]/170	45.8	6	13.9	-	4.8	1.2
Syria [36]/121	36.4	14.9	10.7	-	11.6	2.5
Turkey [40]/1000	7.9	8.9	1.9	21.4	0.9	0.8
Turkey [39]/1058	36.5	32.8	14.1	32.1	4.4	0.9
Egypt [22]/1373	6	38.6	15.8	-	18.1	9.3
Egypt [37]/182	7.8	22.7	15.6	-	34	4.3
Algeria [15]/50	14	12	-	-	50	10
Palestine [35]/504	49	8.5	16.7	-	11.9	1.6
Morocco [14]/120	47	6.5	0	-	32	6.5
Tunisia [13]/139	11.8	7.9	2.1	-	5.7	1.3
Iran [41]/1330	42.4	20.9	18.9	-	2.1	0.2
Iran [42]/130	40.2	17.6	13.7	-	2.4	1.5
